# Knowledge and Practice of Dentists Managing Patients on Antithrombotic Medications: A Cross-Sectional Survey

**DOI:** 10.1055/s-0041-1739436

**Published:** 2022-01-11

**Authors:** Kamis Gaballah, Mawada Hassan

**Affiliations:** 1 Department of Oral and Craniofacial Health Sciences, College of Dental Medicine, University of Sharjah, United Arab Emirates; 2 College of Dentistry, Ajman University, Ajman, United Arab Emirates

**Keywords:** dental practice, antiplatelet, antithrombotics, knowledge, aspirin, clopidogrel

## Abstract

**Objectives** In this article, we explored the level of knowledge and practice of licensed dentists in the United Arab Emirates regarding managing patients on antithrombotic medications. Moreover, to assess the need for educational intervention in this area is one of the objectives.

**Materials and Methods** A total of 502 dentists answered 22 close-ended questionnaires. The sample size was determined based on the registry of the Ministry of Health.

**Results** Only 5.6 and 5.9% showed satisfactory overall awareness about aspirin and plavix, respectively, as drugs may hamper hemostasis. A substantial proportion of dentists consider aspirin (63.1%) and clopidogrel (52.2%) discontinuation before treatment. More than one-third of the participants shall not consider extracting teeth before physician approval, and one-quarter to one-third of them refer patients on such medications to oral surgeons to perform tooth extraction. Most respondents did not adequately answer the questions about the additional hemostatic measures and postoperative analgesia. A significantly high number of participants ( *n* = 440, 87.6%) want to attend updated courses on the dental management of such patients.

**Conclusions** The dentists demonstrate a contrasting diversity of knowledge and practice approaches to patient management on antiplatelet agents. There is an apparent demand to raise understanding of the evidence-based management of a patient on such medications. It is vital to keep formalized training sessions and provide the necessary expertise to students and dentists to prevent unwanted complications.

## Introduction

Dental practitioners commonly encounter patients with chronic illnesses. These patients are often on long-term medications to control these diseases and to prevent their serious complications. The use of oral antithrombotic or antiplatelet agents such as aspirin and clopidogrel as well as anticoagulant such as warfarin has been rising consistently over the last decades to minimize the risk of complications of diabetes, hypertension, ischemic heart diseases, and cerebrovascular diseases. 1 It was estimated that more than 40 percent of the U.S. population older than 40 years receive antiplatelet medications, and nearly one-tenth of U.S. patients are on warfarin. 2

These medications may have significant implications when dentists treat such patients primarily through invasive procedures such as oral surgeries, periodontal procedures, and even block anesthetic injections. The *bounce-back* increase in coagulative activity following the discontinuation of antithrombotic and progressive recovery of platelet aggregation function in addition to decreased fibrinolysis after interrupting aspirin is reported to increase the risk of thromboembolism, 3 4 5 thereby exposing the patient to a potential risk of secondary thrombosis, stroke, and ischemic coronary events. 6 7

The literature is rich in studies investigating dental practitioners' knowledge and awareness about managing patients on various medications. The results are very variable and contradictory. 7 8 9 10 11 This might be related to dental education and training disparities and reinforce the local authorities' good practice guidelines. The knowledge and awareness about managing patients on antithrombotic medication are not an exemption. This study aims to determine the level of knowledge and attitude of dental health care professionals in the United Arab Emirates (UAE) regarding antithrombotic medications and their dental implications and management of patients on such drugs and require dental care. The study was also set to assess the need for additional education and training in this area.

## Materials and Methods

**Fig. 1:**
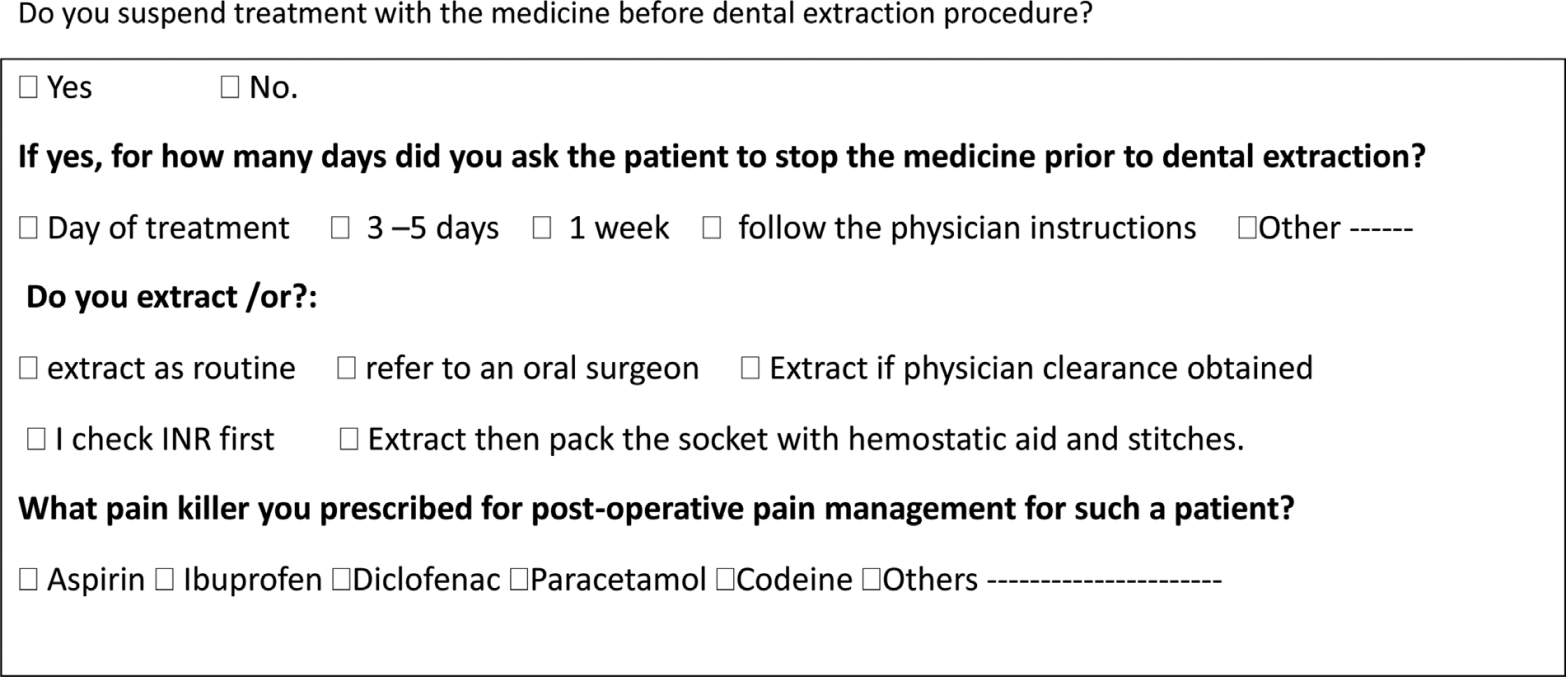
Examples of the survey questions.

This cross-sectional study was conducted to investigate the practicing dentists' awareness about managing patients on antithrombotic agents. A questionnaire was first given to 70 participants in Ajman, Sharjah, and Dubai as part of the pilot study to evaluate and validate the questionnaire to ensure reliability and eliminate confusing questions and vocabularies. The responses were analyzed, and vague questions were either removed or rephrased. The questionnaire's final revision included 22 closed-ended and fixed-choice questions. A group of 502 practicing dentists was then provided with the validated questionnaire. The questionnaire was intended to test dentists' knowledge and attitudes about medications that interfere with thrombosis. The authors aimed to collect information in three main areas: the first, to record the participants' demographic characteristics and the antithrombotic drug cases they treat. The second is the practice of suspending antithrombotic drugs before dental treatment amongst dentists, whether they discontinue it or not or take the physician's consultation along with the standard dental management of such a patient during and after extraction and the appropriate postoperative medications prescribed for such patients. The third part explores the participants' interest in attending training and continuing education courses in the subject. A sample of some of the survey questions is included in Fig. 1 .

Investigators divided the study population into seven strata: Abu Dhabi including the city of Al Ain, Dubai, Sharjah, Ajman, umm al Quwain, Ras Al-Khaimah, and Al Fujairah and then simple random sampling was applied within each stratum independently. The questionnaire was self-administered to practicing dentists in both private and public clinics and hospitals.

### Sample Size Determination

According to the Ministry of Health data, the total number of registered dentists in the UAE is 2,921 dentists distributed over the UAE. A stratification equation was used to determine the appropriate sample size from each emirate 12 :



where *n* = sample size determined by 95% confidence interval and 4% margin of error by the following equation:



A total of 502 participants from different specialties were collected based on stratified random sampling as shown in Table 1 . The data obtained were subjected to descriptive statistical analysis and cross-tabulations using IBM SPSS statistics version 20 software. The *p* -value was calculated with the use of the chi-square test, and the significance was set at below 0.05 at 95 confidence intervals.

**Table 1:** The sample size determination

Stratification procedure		
Emirates	Stratification (ideal sample size)	(What is done)
Abu-Dhabi	( )×500 = 162	160
Dubai	( )×500 = 194	190
Sharjah	( )×500 = 89	83
Ajman	( )×500 = 21	21
Umm Al-Quwain	( )×500 = 5	10
Ras Al Khaimah	( )×500 = 18	25
Al-Fujairah	( )×500 = 11	13
Total	500	502

## Results

**Fig. 2:**
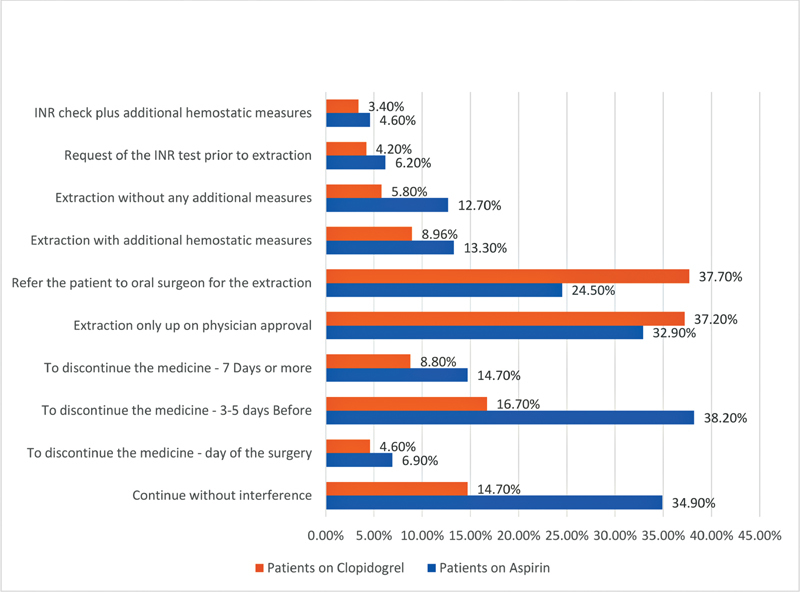
The planning and practice of dental extraction for the patients taking antithrombotic medications. The percentages are calculated from the total number of participants.

Out of 750 forms, 502 were answered entirely, giving a response rate of 66.93%. Only 5.6 and 5.9% showed awareness about aspirin and plavix, respectively, as drugs may hamper hemostasis if regularly taken by their patients. Around a 10th of the participants (12.9%) could not recall any drugs that may compromise the hemostasis. According to the practicing dentists, most of the treated patients (87%) were familiar with these medications' names and effects. Around one-third of individuals on such medications are treated in public hospitals (23.9%) and clinics (7%) as compared with those treated in private hospitals (16.2%) and clinics (12.1%). A substantial proportion of the dentists (63.1 and 52.2%) consider the aspirin and clopidogrel discontinuation before the dental procedure. However, they were not consistent on how long the patient should stop any of the medicines. The remaining 5.1 and 1.9% followed the physician instructions regarding managing patients on aspirin and clopidogrel, respectively. On the other hand, 34.9% do not interfere with aspirin intake, then 14.7% of participants would carry on with treatment without disrupting the clopidogrel intake. The participants' responses to questions related to aspirin and clopidogrel's discontinuation before dental extraction are summarized in Fig. 2 and Table 2 . This study reveals that more than one-third of the participants (32.9%) shall not consider extracting teeth before physician approval, and one-quarter of them (24.5%) refer patients on aspirin and 37.7% of the patients on clopidogrel to oral surgeons to perform the tooth extraction. The additional hemostatic measure in packing the extraction socket with oxidized cellulose was reported by 13.3%. Whereas, 12.7 and 5.8% of participants, while treating the patient on aspirin or clopidogrel, respectively, would extract teeth without additional measures. On the other hand, a relatively high number of dentists ( *n* = 92; 6.2%) request an international normalized ratio test for a patient on aspirin or clopidogrel. Other 3.9% consult the physician and packing the socket with surgicel.

**Table 2:** The pattern of suspension of aspirin and clopidogrel by the different dental disciplines

			Aspirin				Clopidogrel		Total
			Yes	No	Follow physician instruction	Yes	No	Follow physician instruction	
Specialty	GDP	Count	186	109	7	110	46	146	302
		% within specialty	61.6%	36.1%	2.3%	36.4%	15.2%	48.3%	100.0%
		% of total	37.1%	21.7%	1.4%	21.9%	9.2%	29.1%	60.2%
	Oral surgeon	Count	26	14	0	14	13	13	40
		% within specialty	65.0%	35.0%	0.0%	35.0%	32.5%	32.5%	100.0%
		% of total	5.2%	2.8%	0.0%	2.8%	2.6%	2.6%	8.0%
	Endodontist	Count	27	14	0	9	4	28	41
		% within specialty	65.9%	34.1%	0.0%	22.0%	9.8%	68.3%	100.0%
		% of total	5.4%	2.8%	0.0%	1.8%	0.8%	5.6%	8.2%
	Prosthodontist	Count	27	12	2	12	2	27	41
		% within specialty	65.9%	29.3%	4.9%	29.3%	4.9%	65.9%	100.0%
		% of total	5.4%	2.4%	0.4%	2.4%	0.4%	5.4%	8.2%
	Orthodontist	Count	31	14	1	12	6	28	46
		% within specialty	67.4%	30.4%	2.2%	26.1%	13.0%	60.9%	100.0%
		% of total	6.2%	2.8%	0.2%	2.4%	1.2%	5.6%	9.2%
	Others	Count	20	12	0	9	3	20	32
		% within specialty	62.5%	37.5%	0.0%	28.1%	9.4%	62.5%	100.0%
		% of total	4.0%	2.4%	0.0%	1.8%	0.6%	4.0%	6.4%
Total		Count	317	175	10	166	74	262	502
		% within specialty	63.1%	34.9%	2.0%	33.1%	14.7%	52.2%	100.0%
		% of total	63.1%	34.9%	2.0%	33.1%	14.7%	52.2%	100.0%

Abbreviation: GDP, general dental practitioner.

Forty percent of the participants ( *n* = 203) prescribe nonsteroidal anti-inflammatory drugs (NSAIDs) for postoperative pain management. The commonly prescribed NSAIDs were ibuprofen (21.5%), diclofenac (11.2%), and aspirin (7.8%). Paracetamol was considered by 41.1% of the participants, and lastly, codeine was the choice of only 2.4% of the respondents. Approximately 8.6% ( *n* = 43) of all the participants use a combination of pain killers in paracetamol combined with either ibuprofen, diclofenac, or codeine. The remaining 7.2% were not sure about the appropriate pain killer for such a patient.

**Fig. 3:**
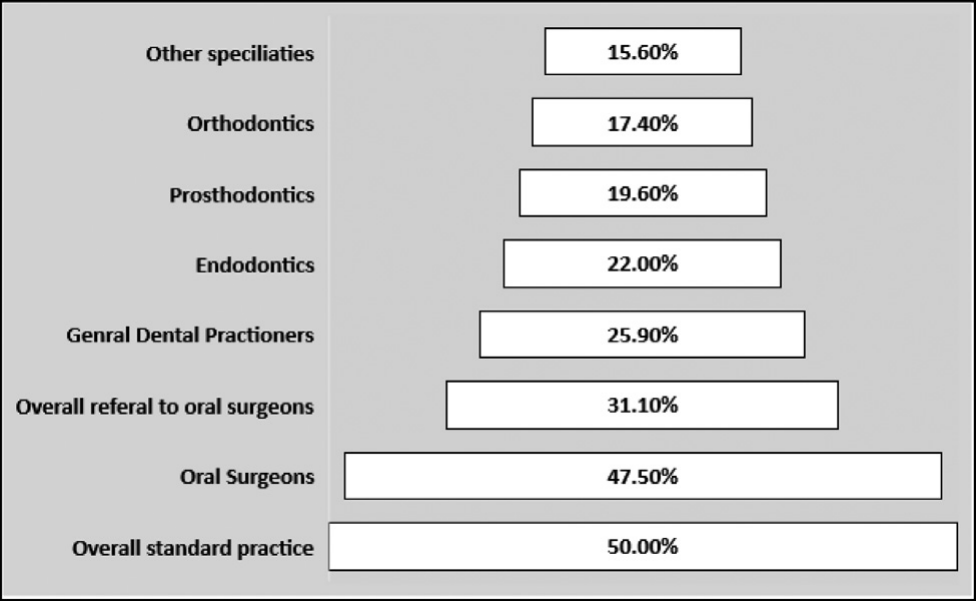
The overall standard practice among the participants and the various dental disciplines.

The overall standard practice among the participants and the various dental disciplines are summarized in Fig. 3 .

A significantly high number of participants ( *n* = 440, 87.6%) want to attend the continuing medical education (CME) course on updating these issues. However, only 62 participants (12.4%) were not interested in attending the CME course on such subjects.

## Discussion

Antithrombotic medications have transformed the management of many severe and life-threatening conditions and can claim a significant increase in survival rates of a patient diagnosed with such diseases. On the other hand, such agents' long-term intake necessitates routine dental surgical patients to overcome the bleeding tendencies attributed to such therapeutics' action. 13 It is estimated that approximately 800,000 people worldwide undergo a nonsurgical coronary artery interventional procedure every year, and most patients with stents are maintained on an antiplatelet regimen. It is therefore prevalent that dental practitioners will encounter these patients regularly. The recent expansion of minimally invasive dentistry may contribute to the practitioners' feeling that their procedures are unlikely to be affected by attending medical history. The worry is that such an approach may affect dentists' awareness and knowledge about medical problems on long-term medications. Research into dental health professionals' knowledge and practice of evidence-based dentistry indicates that awareness of, access to, resource use is variable. 7 8 9 10 11 12 Additionally, the lack of self-confidence due to the deficit in the up-to-date knowledge of the guidelines may influence the dental professional's decision to treat patients with medical conditions. This attitude was noted in the number of referrals to the oral surgeons to perform the procedure or to the physician to assure medical coverage for the dental treatment. The growing litigation against dental malpractice is another stressful factor to consider when investigating the dentist's attitude toward managing patients with complex medical history who require an invasive procedure. 14

Moreover, few general dental practitioners (GDPs) use the internet or electronic databases to influence clinical decisions. 11 The management of patients on antiplatelet drugs requiring extractions in primary dental care may be both inappropriate and inconsistent, 15 as demonstrated by our study, which intends to provide an insight into the current trends for decision-making concerning periprocedural antiplatelet treatment among the practicing dentists in the UAE. Such a goal's practical importance is hard to overestimate since optimal potency and duration of maintenance antiplatelet therapy are still not precise and are barely supported by evidence-based medicine. Therefore, the dentists' poor adherence to the guidelines is familiar and not surprising. 16

This practice is at variance with the current literature, which argues that the interruption of therapy may expose such patients to an increased risk of developing adverse cardiovascular events.

Only a tiny proportion of participants plan to discontinue the medicine in conjunction with the physician's advice. This was in contrast to the Irish and Turkish studies that showed 86 and 90.6% of the dentists, respectively, liaise with physicians any interruption of the antiplatelet medications. 15 17 The decision to interrupt therapy often arrives at the following discussion with the patient's general medical practitioner or cardiologist, a person whose decision may be based on their experience in general surgery or orthopedic surgery. 15 However, the dental practitioner may also decide based on the critical analysis of thorough case history, the current practice's knowledge, and the practitioner's skills to achieve and monitor hemostasis postoperatively.

Our report highlighted the increasing referral to the oral surgeons to perform the extraction and other surgical procedure without any additional communication with the patient medical care team. This trend is based on the assumption that the oral surgeon can assess those patients better and manage any potential complications due to such medications' intake. It is worth mentioning that such an approach might be safer than the general practitioner attempting to extract teeth even in consultation with a physician, as we noted in daily practice. Many of those advised to withdraw the antiplatelet agents for variable periods to facilitate the dentist's job. The oral surgeon's knowledge of the drawback of the aspirin withdrawal and their experience in control and monitor the postextraction bleeding would minimize the unwanted consequences of the regular drug intake disruption. Collet et al investigated 1,358 patients with ischemic heart diseases to relate the disease's severity and mortality to the oral antiplatelet medication intake irregularities. 6 They divided the patient into three groups: never used the drug, prior user, and recently discontinuing group. The latter group was defined as patients who withdraw the antiplatelet agent within the last 3 weeks. The authors concluded that previous users of antiplatelet agents and patients with recent drug interruptions have worse cardiac risks than nonusers. In another study of a cohort of 1,236 patients hospitalized for acute coronary diseases, a total of 51 of these patients discontinued aspirin within 4 weeks of their acute cardiac symptoms. One-third of the latter group withdrawn from aspirin before a dental procedure. The mean time between aspirin's discontinuation and acute cardiac disease ranged from 4 to 17 days. 7

The study has shown that most practitioners were not sufficiently familiar with the standard management and tended to provide suboptimal care for the potential postextraction bleeding. Many dentists may fear that local measures are not sufficient for controlling bleeding in patients with such medications. The literature and practice show that simple local measures in collage sponge or oxidized cellulose packing with hemostatic stitches effectively control postoperative bleeding in patients with antithrombotic or even anticoagulant medications. 18 19 20 21 22 Additionally, several investigators have found that tranexamic acid-soaked gauze, or used as a mouthwash, is quite effective. 13 17

Postoperative pain control is an essential issue in dental practice, especially in patients with concurrent medications. This article showed us the low level of awareness regarding drug interaction with antiplatelet medications. Based on this report, between one-third and one-half of the practitioners prescribe NSAIDs for aspirin or clopidogrel patients. NSAIDs' prescription should be avoided in patients receiving oral antithrombotics because of their additional antiplatelet action and the risk of wound or gastrointestinal hemorrhage.

This article also raised the controversy of prescribing prophylactic antibiotics for patients on oral antiplatelet drugs, and around half of the practitioners prefer to give antibiotics, especially amoxicillin or augmentin, whereas 33.3% disagreed about the idea of prophylactic antibiotics. Patients undergoing dental surgery may be prescribed antibiotics to prevent potential infective endocarditis if the local guidelines recommend treating an existing infection or preventing wound infection in immunocompromised surgical patients. The intake of prophylactic antiplatelet agents per se is not a legitimate indication for the antibiotic prescription. On the other hand, some broad-spectrum antibiotics may increase the risk of bleeding in such patients by disturbing the intestinal flora's balance, leading to decreased vitamin K production required to synthesize the key coagulation factors.

## Conclusion

In conclusion, dentists demonstrate a contrasting diversity of knowledge and practice approaches to patient management on antiplatelet agents. There is an apparent demand to raise understanding of the evidence-based approach to this particular patient population's dental management. It is vital for the dental educational and the governing bodies to set updated local guidelines, organize formal training sessions, and provide the necessary expertise to students and dentists for the safe and effective treatment of such patients.
